# The Therapeutic Effect of Electroconvulsive Therapy on Depressive Patients of Different Ages: A Systematic Review and Meta‐Analysis

**DOI:** 10.1155/da/4345722

**Published:** 2026-08-29

**Authors:** Chifai Tam, Lingling Zhao, Haohao Yan, Ying Peng, Wenbin Guo

**Affiliations:** ^1^ Department of Psychiatry, National Clinical Research Center for Mental Disorders, and National Center for Mental Disorders, The Second Xiangya Hospital of Central South University, Changsha 410011, Hunan, China, csu.edu.cn; ^2^ Department of Psychiatry, National Clinical Research Center for Mental Disorders, The Second Xiangya Hospital of Central South University, No. 139, Renmin Middle Road, Changsha 410011, Hunan, China, csu.edu.cn

**Keywords:** age, electroconvulsive therapy, major depressive disorder, meta-analysis

## Abstract

**Background:**

Major depressive disorder (MDD) has become one of the diseases that seriously affects the quality of life and social function of patients. Electroconvulsive therapy (ECT) is an important method for treating mental disorders. This study aims to conduct a comparison of symptomatic improvement in patients with MDD across different age groups following ECT, thereby providing more precise and personalized treatment plans.

**Methods:**

PubMed, Cochrane Library, CNKI, Wanfang, and other databases were searched for related studies from self‐built databases until February 2025. The retrieved studies were then subjected to a meta‐analysis using R software. Based on the assessment of heterogeneity across studies, either a fixed‐effect model or a random‐effect model was selected for data synthesis.

**Results:**

A total of 15 eligible articles were included in this meta‐analysis, involving 1617 patients with MDD. The results indicated ECT could reduce the scores of the Hamilton Depression Rating Scale (HAMD), Montgomery–Åsberg Depression Rating Scale (MADRS), and Hamilton Anxiety Rating Scale (HAMA). Additionally, it could increase the scores of the Mini‐Mental State Examination (MMSE). However, no statistical significance was observed on the subscales of the Wechsler Memory Scale (WMS). Subgroup analysis and meta‐regression revealed that the older patients (>60 years old) may benefit more significantly in improving depressive symptoms (HAMD and MADRS) and WMS recognition memory. No significant age‐related differences are observed in HAMA, MMSE, or other WMS subtests.

**Conclusions:**

ECT demonstrates therapeutic effects across all age groups of patients with MDD. The older group seemed to benefit more from this treatment modality compared to other age groups. These findings contribute to a better understanding of the differential effects of ECT in patients with MDD of different ages, which may guide clinical decision‐making.

## 1. Introduction

Major depressive disorder (MDD) has emerged as a critical global public health challenge, ranking among the leading causes of disability worldwide. An April 2023 snapshot report by the World Health Organization (WHO) stated that MDD affects an estimated 3.8% of the global population, equivalent to roughly 280 million people [1]. Among adults, the prevalence of the disorder stands at 5%, rising to 5.7% in individuals aged 60 and older. Notably, the age distribution of onset is bimodal: the median age of onset is 26 years in high‐income countries and 24 years in low‐ and middle‐income settings, with a second, more pronounced peak occurring in later life [2]. In China, the lifetime prevalence of MDD reaches 6.8%. Since 2010, MDD has consistently been ranked as the country’s second‐largest contributor to years lived with disability [3].

Despite the widespread use of antidepressants and evidence‐based psychotherapy, some patients fail to respond and develop treatment‐resistant depression. This clinical challenge is amplified in two key populations: adolescents, whose nervous systems are still developing, and older adults, who often manage multiple comorbidities and polypharmacy [4–6].

Electroconvulsive therapy (ECT), a crucial intervention for severe emotional and mental disorders, has been extensively validated for its efficacy and safety [7]. While emerging treatments—including vagus nerve stimulation, transcranial magnetic stimulation, and intranasal esketamine—have been approved for clinical use, the breadth and depth of clinical trials for these newer therapies remain insufficient compared with ECT [2, 3], and they have not yet replaced ECT’s role in depression treatment [6]. Research has demonstrated that ECT can significantly reduce the suicide risk, improve physical functioning and quality of life, and lower hospital readmission rates among patients with MDD.

Age is widely recognized as an important predictor of response to ECT for depression. Two recent meta‐analyses demonstrated that older age is associated with higher rates of response and remission following ECT [8, 9]. However, those studies primarily addressed dichotomous outcomes (response and remission) and did not fully evaluate continuous changes in depressive symptom severity. To address this gap, the present meta‐analysis analyzed continuous outcome measures stratified by age.

However, existing studies have notable limitations:

1. Insufficient age‐stratified research: Most clinical trials lack age‐subgroup analyses, and adolescent patients (<18 years) and elderly patients (>60 years) are often underrepresented, resulting in limited and fragmented efficacy data.
2. Inconsistent efficacy evaluation criteria: Some studies focus solely on alleviating core depressive symptoms while overlooking age‐specific outcomes.
3. Persistent safety concerns: There is a lack of systematic comparisons of ECT‐associated cognitive side effects across different age groups.

Thus, synthesizing global data on ECT efficacy in depressed patients across age groups via systematic review and meta‐analysis and clarifying how age influences treatment outcomes is critical for optimizing clinical decision‐making and developing individualized treatment strategies. While numerous studies have explored the therapeutic effects of ECT, many have focused on specific patient populations or gender‐based comparisons. In contrast, systematic comparative analyses across different age groups remain scarce. In addition, previous studies on cognitive side effects of ECT have used a variety of assessment tools, including multiple tests for memory and executive function in addition to the MMSE and WMS [10, 11]. However, few studies have yet conducted a unified combined analysis of multitool and multidimensional cognitive outcomes [10, 12].

This study aims to systematically search and synthesize domestic and international cohort studies and before‐after‐treatment trials on ECT for depressed patients across different age groups. Using meta‐analytical methods to quantify differences in efficacy and safety, the study focused on two key questions: (1) Whether significant age‐related differences exist in the efficacy of ECT in alleviating core depressive symptoms across adolescent, adult, and elderly patients and (2) the association between age‐related factors and ECT‐associated adverse reactions, such as cognitive impairment. The findings are anticipated to provide evidence‐based support for clinical practice, facilitate the development of precision treatment strategies, and guide treatment selection for depressed patients across age groups.

## 2. Methods

A systematic review and meta‐analysis were performed in accordance with the Preferred Reporting Items for Systematic Reviews and Meta‐Analyses (PRISMA) guidelines. The review protocol was registered at PROSPERO as CRD42025648742.

### 2.1. Search Strategy

Two authors (Chifai Tam and Lingling Zhao) independently identified relevant articles published in PubMed, Cochrane Library, Web of Science, EMbase, China National Knowledge Infrastructure, Wanfang Data, and VIP Database for Chinese Technical Periodicals from inception to February 2025. Taking PubMed as an example, Table 1 lists the terms employed to retrieve 238 studies from this database. The retrieval was filtered by two criteria: study population restricted to humans and publication languages limited to Chinese and English.

**Table 1:** The search strategy of PubMed.

Search strategy	
#1	((((depression[MeSH Terms])OR(Depressive Symptom ^∗^[Title/Abstract]))OR(Symptom,Depressive[Title/Abstract]))OR(Emotional Depression[Title/Abstract]))OR(Depression,Emotional[Title/Abstract])
#2	((((((((((Electroconvulsive Therapies[MeSH Terms])OR(Therap ^∗^, Electroconvulsive[Title/Abstract]))OR(Shock Therap ^∗^ Electric[Title/Abstract]))OR(Electric Shock Therap ^∗^[Title/Abstract]))OR(Therap ^∗^,Electric Shock[Title/Abstract]))OR(ECT (Psychotherapy[Title/Abstract]))OR(Convulsive Therap ^∗^,Electric[Title/Abstract]))OR(Electric Convulsive Therap ^∗^[Title/Abstract]))OR(Therap ^∗^,Electric Convulsive[Title/Abstract]))OR(Electroshock Therap ^∗^[Title/Abstract]))OR(Therap ^∗^,Electroshock[Title/Abstract])
#3	(((((((((((((Treatment Outcome ^∗^[Mesh])OR(TreatmentEfficacy[Title/Abstract]))OR(Efficacy, Treatment[Title/Abstract]))OR(Clinical Efficacy[Title/Abstract]))OR(Efficacy,Clinical[Title/Abstract]))OR(Rehabilitation Outcome[Title/Abstract]))OR(Outcome,Rehabilitation[Title/Abstract]))OR(Clinical Effectiveness[Title/Abstract]))OR(Effectiveness, Clinical[Title/Abstract]))OR(Treatment Effectiveness[Title/Abstract]))OR(Effectiveness,Treatment[Title/Abstract]))OR(Patient‐Relevant Outcome[Title/Abstract]))OR(Outcome ^∗^,Patient‐Relevant[Title/Abstract]))OR(Patient Relevant Outcome ^∗^ [Title/Abstract])
#4	((((((((((((Adolescent ^∗^[Title/Abstract])OR(Adolescence[Title/Abstract]))OR(Youth ^∗^[Title/Abstract]))OR(Teen ^∗^[Title/Abstract]))OR(Teenager ^∗^[Title/Abstract]))OR(Adult ^∗^[Title/Abstract]))OR(Pediatrics[Title/Abstract]))OR(Elderly[Title/Abstract]))OR(Aged[Title/Abstract]))OR(Age Group ^∗^[Title/Abstract]))OR(young‐old[Title/Abstract]))OR(old‐old[Title/Abstract]))OR(child ^∗^[Title/Abstract])
#5	#1 AND #2 AND #3 AND #4

### 2.2. Study Selection Criteria

Studies were included if patients were diagnosed with depression based on criteria such as the Diagnostic and Statistical Manual of Mental Disorders (DSM‐IV), the Chinese Classification of Mental Disorders, 3rd Edition (CCMD‐3), or the International Classification of Diseases (ICD‐10), with no specific requirements for patients’ age, disease duration, or other factors. In addition, studies were included if they used ECT as the intervention. The primary outcome measures are the Hamilton Depression Scale (HAMD) and the Montgomery–Åsberg Depression Rating Scale (MADRS). The secondary outcome measures include the Hamilton Anxiety Scale (HAMA), the Mini‐Mental State Examination (MMSE), and the Wechsler Memory Scale (WMS).

Non‐Chinese and non‐English literature, studies without specified outcome measures, original studies with incomplete data or inaccessible full texts, animal experiments, and literature involving the combined use of two or more nonpharmacological treatments were excluded.

### 2.3. Data Extraction and Quality Assessment

Two authors (Chifai Tam and Lingling Zhao) independently extracted the following data from the included articles: the name of the first author; publication dates; the countries or regions; study design; participants; diagnosis guidelines; interventions; intervention duration; sample sizes for different age groups and total sample size; sex ratio, disease duration, educational level, and marital status of subjects in different age groups; scales used for assessment; and mean scores and standard deviations before and after treatment.

Two authors (Chifai Tam and Lingling Zhao) independently evaluated the risk of bias of the included studies. The Newcastle–Ottawa Scale (NOS) was used to assess the quality of studies included in the meta‐analysis. The NOS consists of three dimensions: selection of study subjects (0–4 points), comparability between groups (0–2 points), and outcome measurement (0–3 points), with a total score of 9 points. A higher score indicates a better quality.

### 2.4. Data Analysis

Data analyses were performed by R software for meta‐analysis and publication bias analysis. For meta‐regression analysis, the regression coefficient (*β*), 95% confidence intervals (CI), and *p*‐value were used to quantify the extent of age’s influence on the effect size and statistical significance. Cohort studies and before‐after‐treatment studies were conducted separately due to fundamentally different effect measures, variance structures, and bias profiles. Subgroup analysis was performed based on mean change and SD change scores, assuming a pre–post correlation of *r* = 0.6 for variance calculation. For subgroup analysis, the standardized mean difference (SMD) expressed with a 95% CI was used as the effect‐size indicator. Heterogeneity among studies was assessed via a chi‐square test. As recommended by the Cochrane Handbook and PRISMA guidelines, a random‐effects model was applied for all meta‐analyses.

## 3. Results

### 3.1. Literature Search

**Figure 1:**
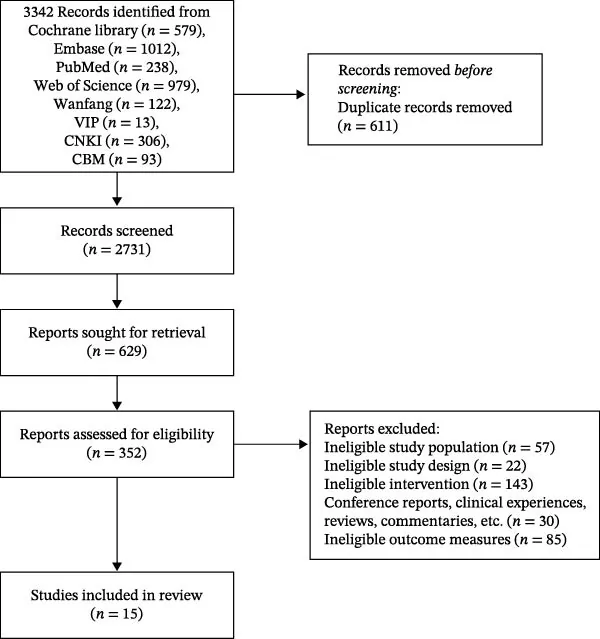
Selection procedures of studies.

Our initial search identified 3342 records. A total of 611 articles were duplicates. After duplicates were removed, 2731 studies were excluded based on their titles and abstracts. A total of 352 potentially relevant records were retrieved for detailed full‐text evaluation. Finally, 15 articles [13–27] met the selection criteria and were deemed to contain data relevant to the systematic review and meta‐analysis. A PRISMA diagram detailing the article selection process is shown in Figure 1.

### 3.2. Characteristics of Included Studies

A total of 1617 people participated and were included across the 15 articles. Among these, six studies were cohort studies and nine were before‐after‐treatment studies. The quality of the included studies was assessed using the NOS, with three articles scoring 7 points, eight articles scoring 8 points, and four articles scoring 9 points. Table 2 shows the basic characteristics of the included articles as well as the results of the bias risk assessment. Some data were missing from the included articles. No feedback was received after emailing the corresponding authors. As the missing data did not affect the meta‐analysis, we used the existing data to conduct the analysis.

**Table 2:** Basic features of included studies.

Study	Research design	Age (mean ± SD), *N*, Sex (M/F)	Outcome measure	NOS scale/points
Sarma et al. [13]	Cohort study	44.82 ± 27.58, *n* = 126, 47/79 74.02 ± 15.66, *n* = 184, 57/127	③	7
Li et al. [15]	Cohort study	15.51 ± 1.6, *n* = 110, 42/68 32.18 ± 12.8, *n* = 100, 41/59	②	8
Xu et al. [14]	Before–after‐treatment study	22.0 ± 3.9, *n* = 41, 17/24	①③	9
Wang [16]	Cohort study	15.4 ± 1.2, *n* = 19, 1/18 26.8 ± 12.8, *n* = 28, 13/15	①②	7
Heijnen et al. [17]	Cohort study	—, *n* = 13, 5/8 —, *n* = 50, 19/31 —, *n* = 33, 8/25	②	7
Socci et al. [18]	Cohort study	21.2 ± 6, *n* = 137, 63/74 32.7 ± 13.2, *n* = 73, 57/116 39.5 ± 17.8, *n* = 92, 19/73	②④	8
Pu and Liao [20]	Before–after‐treatment study	36.12 ± 5.35, *n* = 50, 39/11	②	8
Liang [21]	Before–after‐treatment study	54.5 ± 3.6, *n* = 180, 79/101	①②④	9
Bouckaert et al. [22]	Before–after‐treatment study	71.9 ± 7.8, *n* = 28, 8/20	③④	8
Lou et al. [25]	Before–after‐treatment study	32.96 ± 9.42, *n* = 42, 19/26	②⑤	8
Birkenhäger et al. [26]	Cohort study	38.5 ± 5.9, *n* = 33, 9/24 55.2 ± 5.4, *n* = 76, 20/56 74.0 ± 6.0, *n* = 32, 7/25	②	9
Zhao et al. [24]	Before–after‐treatment study	68.7 ± 5.60, *n* = 45, 21/24	①②④⑤	8
Zhang [19]	Before–after‐treatment study	42.07 ± 7.57, *n* = 30, 17/13	②⑤	9
Xu et al. [27]	Before–after‐treatment study	67.30 ± 46.00, *n* = 49, —	②⑤	8
Xie et al. [23]	Before–after‐treatment study	65.00 ± 4.80, *n* = 43, 18/25	①②④	8

*Note:* ① = HAMA, ② = HAMD, ③ = MADRS, ④ = MMSE, ⑤ = WMS.

### 3.3. Meta‐Analysis

The meta‐analysis of scores of HAMD, MADRS, HAMA, MMSE, and WMS was performed according to age. The meta‐analysis of cohort studies and before‐after‐treatment studies was conducted using R software, respectively.

### 3.4. Depressive Symptoms

**Figure 2:**
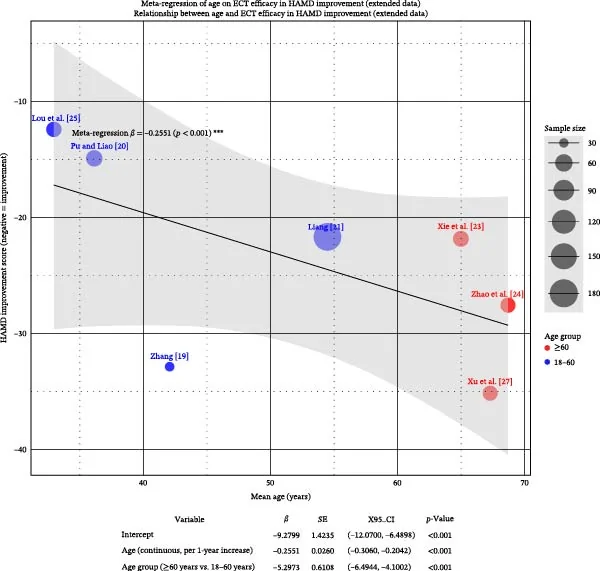
Meta‐regression of age on ECT efficacy in HAMD improvement.

HAMD score data were provided in 12 studies [15–21, 23–27]. Meta‐regression analysis of HAMD revealed that each additional year of age was associated with a greater reduction in HAMD scores (*β* = −0.255, 95% CI: −0.306 to −0.204, *p* < 0.001, Figure 2).

**Figure 3:**
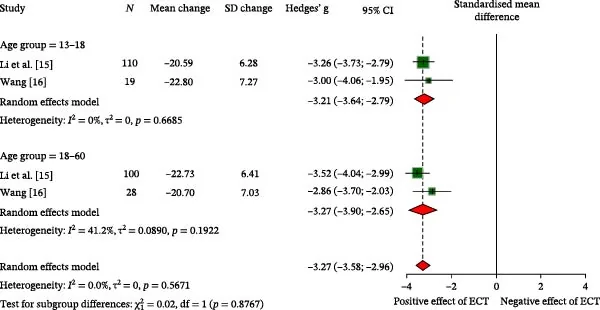
Forest plot of cohort studies: ECT effect on HAMD scores in depressed patients (13–18 and 18–60 years old).

**Figure 4:**
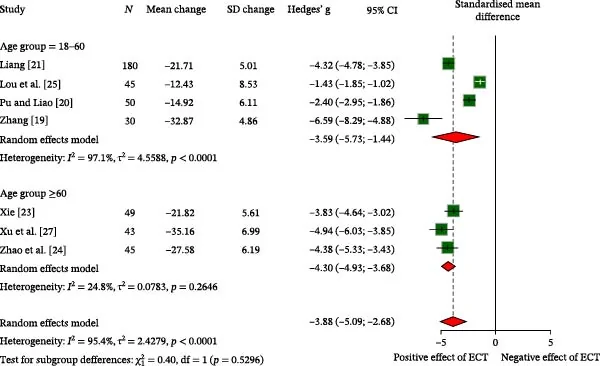
Forest plot of before‐after‐treatment studies: ECT effect on HAMD scores in depressed patients (18–60 and >60 years old).

ECT exerted a significant effect on reducing HAMD scores in depressed patients across the age groups of 13–18 18–60 and over 60 years. Specifically, the combined effect sizes were SMD = −3.27 (95% CI: −3.58 to −2.96, *p* < 0.05, *I* ^2^ = 0%, Figure 3) for the 13–18 and 18–60 years groups and SMD = −3.88 (95% CI: −5.09 to −2.68, *p* < 0.05, *I* ^2^ = 95.4%, Figure 4) for the 18–60 and over 60 years groups.

**Figure 5:**
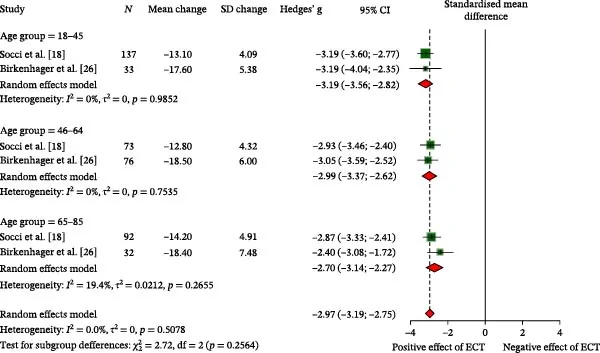
Forest plot of cohort studies: ECT effect on HAMD scores in depressed patients (18–45, 46−64, and 65–85 years old).

A meta‐analysis of two studies was conducted separately due to overlapping age groupings across the other studies and the lack of raw patient‐level data. ECT had a significant effect on reducing HAMD scores in patients with depression who were ≤45 years, 46–64 years, and 65–85 years (SMD = −2.97, 95% CI = −3.19 to −2.75, *p* < 0.05, *I* ^2^ = 0%, Figure 5).

Subgroup analysis for other patients was not conducted in W. T. C. J. Heijnen’s study [17] due to limited data. ECT had a significant effect on reducing HAMD scores in patients with depression (SMD = −2.34, 95% CI = −2.73 to −1.95, *p* < 0.05). The mean reduction (± SD) in HAMD scores for each age group was 18.3 ± 9.5 (<50 years old), 22.3 ± 8.8 (51–67 years old), and 21.1 ± 9.2 (>69 years old), respectively.

The MADRS score data were provided in three studies [13, 14, 22].

**Figure 6:**
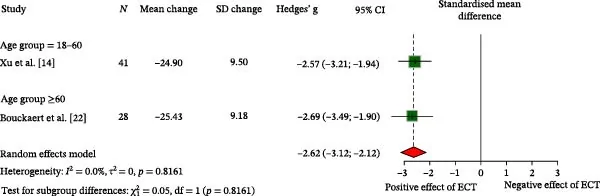
Forest plot of before‐after‐treatment studies: ECT effect on MADRS scores in depressed patients (18–60 and >60 years old).

**Figure 7:**
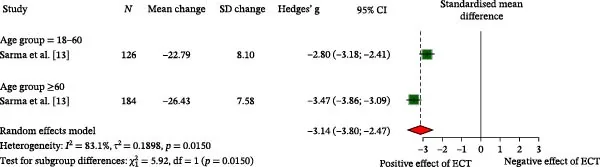
Forest plot of cohort studies: ECT effect on MADRS scores in depressed patients (18–60 and >60 years old).

ECT exerted a significant effect on reducing MADRS scores in depressed patients aged 18–60 and over 60 years. Specifically, the combined effect sizes were SMD = −2.62 (95% CI: −3.12 to −2.12, *p* < 0.05, *I* ^2^ = 0%, Figure 6) and SMD = −3.14 (95% CI: −3.80 to −2.47, *p* < 0.05, *I* ^2^ = 83.1%, Figure 7) for the same age groups from cohort studies and before‐after‐treatment studies, respectively.

### 3.5. Anxiety Symptoms, Mental State, and Cognitive Function

**Figure 8:**
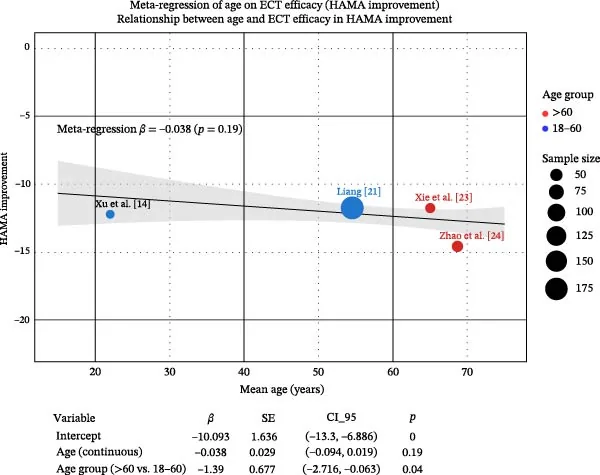
Metaregression of age on ECT efficacy in HAMA improvement.

HAMA score data were provided in four studies [14, 21, 23, 24]. Meta‐regression analysis of HAMA showed a nonsignificant trend toward age moderation of ECT efficacy. Each additional year of age was associated with a nominal increase in anxiety symptom improvement (*β* = −0.038, 95% CI: −0.094 to 0.019, *p* = 0.19, Figure 8).

**Figure 9:**
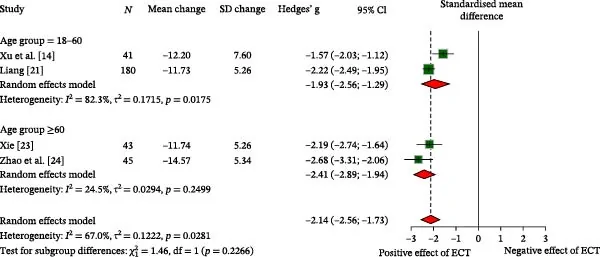
Forest plot of before–after‐treatment studies: ECT effect on HAMA.

ECT had a significant effect on reducing HAMA scores in patients with depression who are 13–18 years old and over 60 years old (SMD = −2.14, 95% CI = −2.56 to −1.73, *p* < 0.05, *I* ^2^ = 67%, Figure 9).

**Figure 10:**
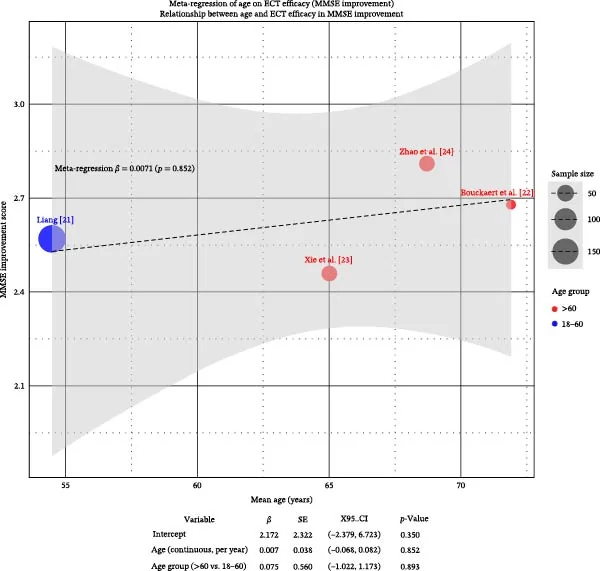
Metaregression of age on ECT efficacy in MMSE improvement.

MMSE score data were provided in four studies [21–24]. Meta‐regression analysis of MMSE showed a nonsignificant trend toward age moderating the effect of ECT on global cognition. Each additional year of age corresponded to a negligible 0.007 point change in MMSE score (*β* = 0.007, 95% CI: −0.068 to 0.082, *p* = 0.852, Figure 10).

**Figure 11:**
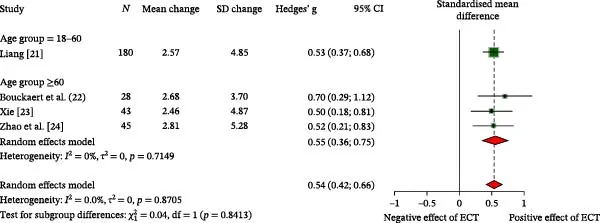
Forest plot of before–after‐treatment studies: ECT effect on MMSE.

ECT had a significant effect on improving MMSE scores in patients with depression who were 18–60 years old and over 60 years old (SMD = 0.54, 95% CI = 0.42 to 0.66, *p* < 0.05, *I* ^2^ = 0%, Figure 11).

**Figure 12:**
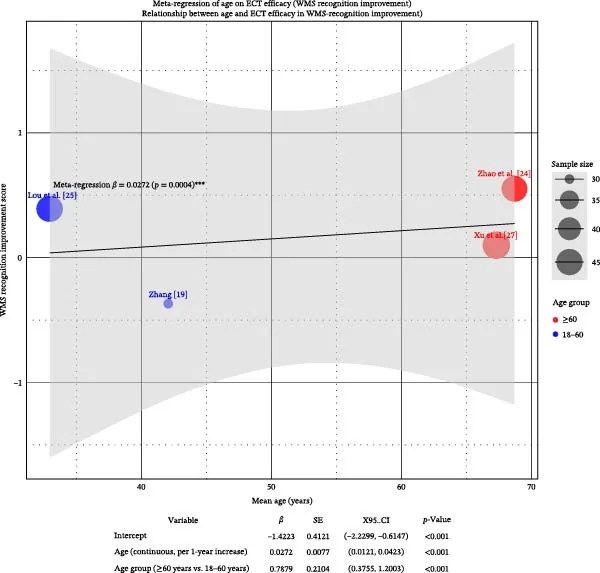
Metaregression of age on ECT efficacy in WMS‐recognition improvement.

The WMS score data were provided in four studies [19, 24, 25, 27]. Meta‐regression analysis of WMS‐Recognition revealed that each additional year of age was associated with a greater improvement in recognition memory scores (*β* = 0.272, 95% CI: 0.012 to 0.042, *p* < 0.001, Figure 12).

**Figure 13:**
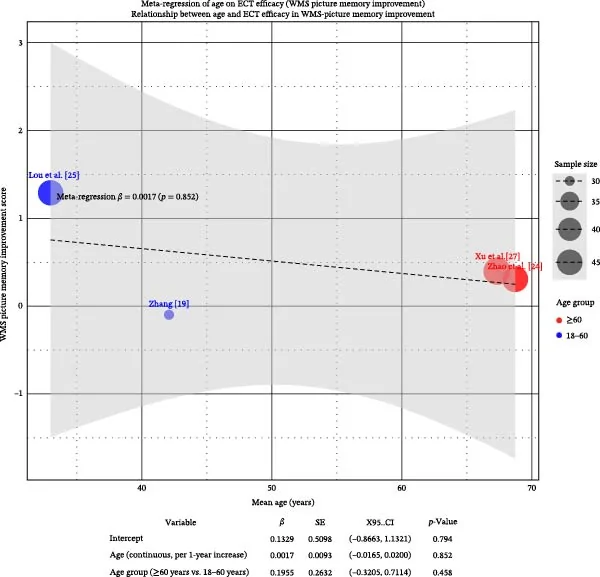
Metaregression of age on ECT efficacy in WMS‐picture improvement.

Meta‐regression analysis of the WMS‐Picture showed no significant moderating effect of age. The continuous age coefficient was *β* = 0.002 (95% CI: −0.017 to 0.020, *p* = 0.852, Figure 13), and the categorical comparison yielded *β* = 0.196 (95% CI: −0.321 to 0.711, *p* = 0.458).

**Figure 14:**
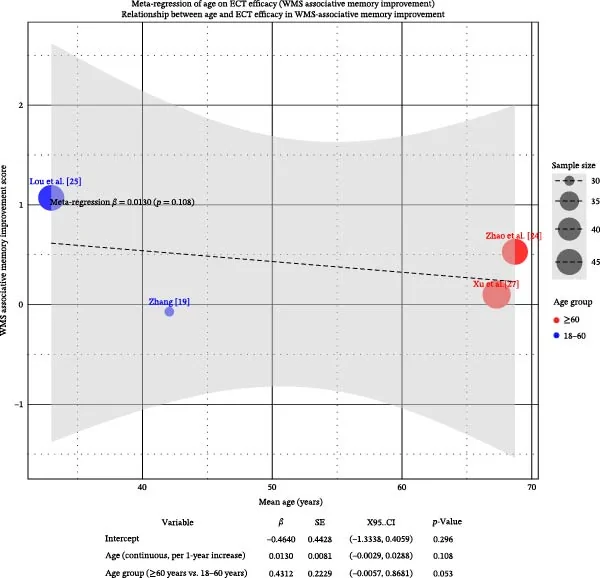
Metaregression of age on ECT efficacy in WMS‐association improvement.

A marginally significant positive trend was observed in the meta‐regression analysis of WMS‐Association. The continuous age analysis showed *β* = 0.013 (95% CI: −0.003 to 0.029, *p* = 0.108, Figure 14), while the categorical comparison approached statistical significance (*β* = 0.431, 95% CI: −0.006 to 0.868, *p* = 0.053).

**Figure 15:**
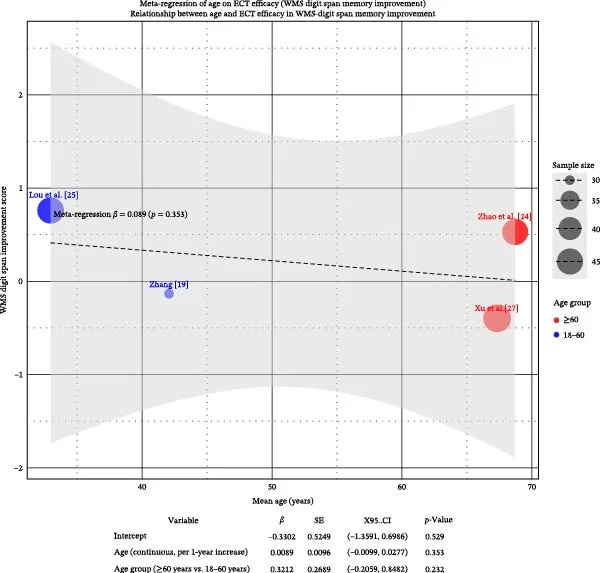
Metaregression of age on ECT efficacy in WMS‐digitspan improvement.

Meta‐regression analysis of WMS‐Digitspan revealed no significant age moderation. The results were *β* = 0.009 (95% CI: −0.010 to 0.028, *p* = 0.353, Figure 15) for continuous age and *β* = 0.321 (95% CI: −0.206 to 0.848, *p* = 0.232) for categorical comparison.

**Figure 16:**
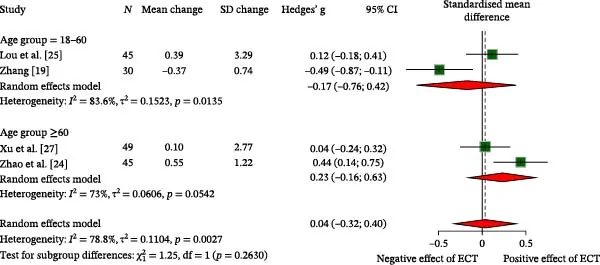
Forest plot of before–after‐treatment studies: ECT effect on WMS‐Recognition.

**Figure 17:**
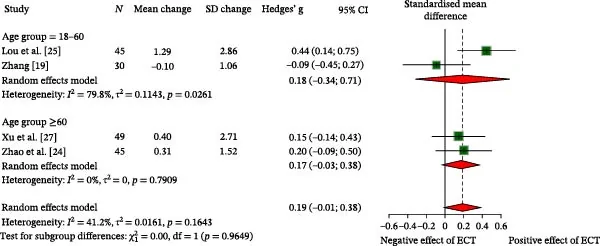
Forest plot of before–after‐treatment studies: ECT effect on WMS‐pictures scores.

**Figure 18:**
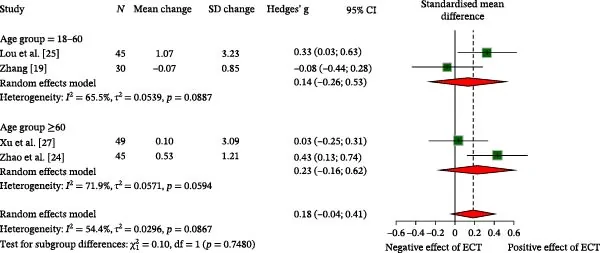
Forest plot of before–after‐treatment studies: ECT effect on WMS‐association.

**Figure 19:**
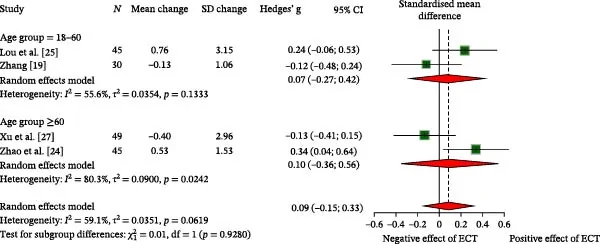
Forest plot of before–after‐treatment studies: ECT effect on WMS‐digitspan.

ECT had no significant improvement or damaging effect on WMS‐Recognition (SMD = 0.04, 95% CI = −0.32 to 0.40, *p* > 0.05, *I* ^2^ = 78.8%, Figure 16), WMS‐Pictures (SMD = 0.19, 95% CI = −0.01 to 0.38, *p* > 0.05, *I* ^2^ = 41.2%, Figure 17), WMS‐Association (SMD = 0.18, 95% CI = −0.04 to 0.41, *p* > 0.05, *I* ^2^ = 54.4%, Figure 18), and WMS‐Digitspan (SMD = 0.09, 95% CI = −0.15 to 0.33, *p* > 0.05, *I* ^2^ = 59.1%, Figure 19).

### 3.6. Sensitivity Analysis

Sensitivity analysis was conducted by changing the effect model. The results of the heterogeneity tests across all trials remained unchanged after the effect model was modified.

Primary sources of heterogeneity were identified by removing the included studies.

For meta‐regression analysis, sensitivity analyses were conducted for the two significant moderating effects (HAMD and WMS‐Recognition, Table 3). Detailed results of the sensitivity analyses are provided in the Supporting Information. After removing any single HAMD study, the *β* values remained negative (range: −0.163 to −0.419), with statistical significance maintained (all *p* < 0.001). Varying the pre–post correlation assumption (*r* = 0.5, 0.6, and 0.7) did not alter the β estimate (−0.255). After removing Zhang [19] or Zhao et al. [24] from the WMS‐Recognition analysis, the effect was not significant (*p* = 0.837 and 0.585, respectively).

**Table 3:** Sensitivity analysis of meta‐regression analysis.

Measure	Leave‐one‐out robustness	Correlation (*r* = 0.5–0.7) robustness	Overall conclusion
HAMD	Robust (All *p* < 0.001)	Robust (*β* unchanged)	Highly robust
WMS‐Recognition	Partially robust (2/4 exclusions: *p* > 0.05)	Robust (*β* unchanged)	Partially robust–limited by small *k* (*n* = 4)

However, the fixed‐effect model of WMS‐Picture and WMS‐Association exhibited a statistical improvement (*p* < 0.05), showing that the results are subject to the choice of statistical model (Table 4). The lack of awareness of fixed‐effects models regarding between‐study heterogeneity might explain this discrepancy. Therefore, the significant results of the fixed‐effects model should be regarded with caution. For subgroup analysis, after removing studies on HAMD, MADRS, MMSE, and WMS‐Digitspan, no source of heterogeneity was identified. The combined results were robust. For the HAMA scale, excluding Xu Shuxian’s study reduced *I* ^2^ from 67% to 2.8%. For the WMS‐Recognition scale, excluding Zhang Qi’s study reduced *I* ^2^ from 78.8% to 52%. For the WMS‐Pictures scale, excluding Lou Dandan’s study reduced *I* ^2^ from 59.1% to 0%, while excluding Zhang Qi’s study reduced *I* ^2^ from 59.1% to 10.78%. For the WMS‐Association scale, excluding Lou Dandan’s study reduced *I* ^2^ from 54.4% to 0%. The combined results were robust.

**Table 4:** Sensitivity analysis of subgroup analysis.

Outcome measure	Heterogeneity test		Effect model	SMD	95% Cl	*p*‐Value
	*p*‐Value	*I* ^2^ (%)				
HAMD (Figure 3)	0.5671	0	Fixed	−3.27	−3.58, –2.96	<0.05
HAMD (Figure 4)	<0.0001	95.4	Fixed	−3.03	−3.27, –2.79	<0.05
HAMD (Figure 5)	0.5078	0	Fixed	−2.97	−3.19, –2.75	<0.05
HAMA	0.0281	67	Fixed	−2.14	−2.34, –1.93	<0.05
MMSE	0.8705	0	Fixed	0.54	0.42, 0.66	<0.05
WMS‐Recognition	0.0027	78.8	Fixed	0.07	−0.08, 0.23	>0.05
WMS‐Pictures	0.1643	41.2	Fixed	0.19	0.04, 0.34	<0.05
WMS‐Association	0.0867	54.4	Fixed	0.19	0.04, 0.34	<0.05
WMS‐Digitspan	0.0619	59.1	Fixed	0.09	−0.06, 0.24	>0.05

### 3.7. Publication Bias

Funnel plot inspection combined with Egger’s test was used to assess whether publication bias existed. The funnel plots revealed a roughly symmetric distribution of the study. Egger’s test did not identify any substantial publication bias with *p* > 0.05. These findings suggested that the meta‐analysis was unlikely to be affected by publication bias. However, due to limited data, the statistical power of the publication bias analysis using funnel plots and Egger’s test was inadequate. The results should be interpreted cautiously.

## 4. Discussion

The findings of this study indicate that ECT reduces scores on the HAMD, MADRS, and HAMA across all age groups of patients with depression while increasing scores on the MMSE. These findings suggest that ECT may exert a positive effect on depressive and anxiety symptoms.

The older group (≥60 years) in the HAMD showed a larger mean improvement of 5.30 points (*β* = −5.297, 95% CI: −6.494 to −4.100, *p* < 0.001). The analysis indicates that advancing age appears to be a positive predictor of the antidepressant response to ECT. These results suggest that patients aged 60 years and above may derive a greater benefit than the younger patients.

The pooled SMD across all subgroups in the forest plot for HAMD and MADRS was negative, indicating that ECT has therapeutic effects on MDD. The significant absolute value of SMD suggests that ECT may significantly improve depressive symptoms in patients with depression across various age groups. The pooled SMD across the >60 years old group for both HAMD and MADRS was slightly greater than that across the 18–60 years old group. However, the subgroup differences in *p*‐values for HAMD were larger than 0.05, suggesting that age may not be a key factor in ECT efficacy. There may not be a statistically significant difference in HAMD scores for ECT efficacy across age groups. The subgroup differences in *p*‐values for MADRS were less than 0.05, suggesting that elderly patients may show greater efficacy on MADRS scores after ECT.

The association between each additional year of age and the improvement in HAMA scores did not reach statistical significance (*p* = 0.19). The categorical comparison between older people (≥60 years) and adults (18–60 years) showed a difference of −1.390 points (*β* = −1.390, 95% CI: −2.716 to −0.063, *p* = 0.04).

The pooled SMD across all subgroups in the forest plot for HAMA is negative, indicating that ECT has therapeutic effects on MDD. The significant absolute value of SMD suggests that ECT may significantly improve anxiety symptoms in patients with depression across various age groups. The subgroup differences in the *p*‐value for HAMA were larger than 0.05, suggesting that age may not be a key factor in ECT efficacy. There may not be a statistically significant difference in HAMA scores for ECT efficacy across age groups.

For the meta‐analysis of MMSE and WMS data, we included measurements obtained after patients had completed at least six ECT treatments (≥2 weeks of treatment).

The association between each additional year of age and the improvement in HAMA scores appeared to be clinically irrelevant (*p* = 0.19). The categorical comparison between older people (≥60 years) and adults (18–60 years) in MMSE showed a nonsignificant difference of 0.075 points (*β* = 0.075, 95% CI: −1.022 to 1.173, *p* = 0.458). The older people (≥60 years) showed a larger mean improvement only in WMS‐Recognition (*β* = 0.788, 95% CI: 0.376 to 1.200, *p* < 0.001). Among the WMS subtests, recognition memory demonstrated the most substantial age‐related enhancement following at least 2 weeks of treatment, with older adults showing greater improvement compared to that of younger adults. Picture memory and digit span showed no significant age effects, while associative memory displayed a marginally positive trend. Notably, the timing of cognitive assessment varied considerably across studies, with acute impairment commonly observed immediately after ECT and improvement more frequently reported at a longer follow‐up; such heterogeneity might bias the apparent association between advanced age and greater cognitive benefits following ECT [28].

Using a random‐effect model, the pooled SMD showed no significant changes in MMSE and WMS scores following ECT. The *p*‐values of the subgroup differences were greater than 0.05, suggesting that age may not be a key factor in ECT efficacy. There might not be a statistically significant difference in ECT efficacy among various age groups. Furthermore, ECT might not substantially impair or enhance cognitive function, recognition memory, picture memory, associative memory, or digit span memory in patients with depression after at least two weeks of treatment. Additionally, no age‐related differences in cognitive impairment were observed across the subgroups.

Sensitivity analyses of meta‐regression confirmed that the significant moderating effect of advancing age on ECT efficacy, particularly for depressive symptoms (HAMD), was a highly robust finding. The association remained statistically significant regardless of which individual study was excluded or which pre–post correlation coefficient was assumed. This reinforces the conclusion that ECT may exhibit superior antidepressant efficacy in older adults. However, the positive moderating effect on WMS recognition memory was contingent on specific studies due to the limited number of trials. Therefore, further studies are required to determine whether ECT is potentially effective in enhancing specific memory domains in the elderly.

Sensitivity analyses of the subgroup suggested that the studies by Zhang Qi and Xu Shuxian introduced bias in the estimation of the effect size, and their research results might have deviated from the true effect size. The SMD = 2.79 of Xu Shuxian’s study was significantly higher than that of the other included studies. Its abnormally large effect size might be the core reason for the increased heterogeneity. Zhang Qi’s study conducted 12 ECT treatments, while the other studies conducted only 6–8 treatments. The number of treatments might lead to heterogeneity and effect‐size discrepancies. Despite conducting a thorough review of Lou Dandan’s study methodology and confirming the accuracy of data extraction and calculation, no inconsistency with other studies was found. There might be other confounding factors in the research process that would affect the accurate assessment of the therapeutic effect of ECT.

In conclusion, the sensitivity analysis indicates that the results of the meta‐analysis were relatively stable and reliable.

The results of this study are consistent with those of other similar studies, which show that ECT is generally effective for patients with depression. Based on the neural plasticity hypothesis, the therapeutic mechanism of ECT involves enhancing neural plasticity and causing structural changes in the brain [29]. ECT regulates the neurotransmitter system, for example, by improving the activity and concentration of neurotransmitters such as serotonin, dopamine, and norepinephrine. Serotonin is closely related to emotion regulation, dopamine engages the reward mechanism and emotional regulation, and norepinephrine impacts attention and arousal levels. Meanwhile, ECT promotes the formation of new connections and synapses in neurons in brain regions closely related to emotion regulation, enhances the functional connections between these brain regions, repairs abnormal neural pathways caused by depression, and thereby improves emotional symptoms.

This study explores the efficacy of ECT on patients of different age groups through meta‐analysis, including minors (13–18 years old) [30], adults (18–60 years old), and the elderly (over 60 years old) [31] with depression. The meta‐analysis indicates that the overall efficacy of ECT for the elderly appeared to be slightly better than that of other age groups with depression. Therefore, this study suggests that ECT may yield more pronounced therapeutic benefits in older patients with depression. Following a comprehensive assessment of the patient’s neurological status, as well as cardiovascular, respiratory, hepatic, and renal functions, those deemed tolerant and with clear indications might undergo ECT. Concurrently, the dosage and administration of other medications should be adjusted accordingly. The decision to prioritize ECT should be based on clinical circumstances to optimize treatment outcomes.

## 5. Limitations

The limitations of this study are as follows: (1) limited sample size: only 15 articles were included, with a relatively small total sample size, leading to large sampling errors. In age‐stratified analyses, insufficient data failed to clearly demonstrate the trend of efficacy changes and the relationship between age and efficacy. (2) Statistical analysis: the results showed high heterogeneity, making the pooled outcomes difficult to interpret accurately. In subgroup analyses, the limited sample size prevented full coverage of all potential efficacy‐influencing factors, risking the omission of important information and reducing reliability. (3) Other influencing factors: variations in ECT protocols among original studies, including treatment frequency, current intensity, and combination with other therapies, might lead to differences in final efficacy, thereby reducing the accuracy of efficacy assessment. Furthermore, this study primarily analyzed ECT efficacy in depressed patients of different ages at the overall level, failing to fully consider individual differences such as genetic factors, life experiences, and psychological states, which to some extent affect the generalizability and accuracy of the conclusions.

## 6. Conclusions

ECT significantly improves depressive (HAMD and MADRS) and anxiety (HAMA) symptoms in depressed patients of all ages. After at least 2 weeks of treatment, there are no apparent adverse effects on global cognition (MMSE) or memory (WMS), indicating favorable cognitive safety. Age exerts a selective moderating effect on ECT efficacy. Older patients appear to benefit more in terms of depressive symptoms and WMS recognition memory enhancement. No significant age‐related differences are observed in the HAMA, MMSE, and other WMS subtests. Sensitivity analyses and the absence of publication bias confirm the stability and reliability of these findings.

## Author Contributions

Chifai Tam and Lingling Zhao designed the study, performed the research, collected and analyzed the data, drafted the manuscript, and conducted the statistical analysis. Haohao Yan and Wenbin Guo contributed to the conception and design. Ying Peng contributed to the statistical analysis and interpretation of data as well as supervision during the revision. Haohao Yan, Ying Peng, and Wenbin Guo made improvements to the manuscript. All of the authors contributed to the final work.

## Funding

This study was supported by the 2024 Undergraduate Innovation and Entrepreneurship Training Program Support Project (Grant CXPY2024248).

## Disclosure

We would like to state that no persons or third‐party services involved in the research or manuscript preparation are listed as authors and have not been acknowledged. The entire text was contributed by the authors listed. It should be noted that parts of the manuscript preparation were all completed manually, such as data extraction, chart production, and so on. After DeepSeek generated the refined English text, the authors compared it with the original draft sentence by sentence. The authors ensured that the scientific accuracy, logical coherence, and intended meaning of the content were maintained. The authors carefully considered whether each proposed change for Grammarly’s and QuillBot’s grammatical checks was appropriate in the given context and approved only those that improved the manuscript’s correctness and clarity without altering its scientific content. The funder had no role in study design, data collection, analysis, decision to publish, or preparation of the manuscript.

## Conflicts of Interest

The authors declare no conflicts of interest.

## Supporting Information

Additional supporting information can be found online in the Supporting Information section.

## Supporting information


**Supporting Information** Since this study is a meta‐analysis, we did not directly use the MMSE in our research. Instead, we extracted data related to the MMSE from the included primary studies. These primary studies, which had already obtained the necessary permissions for using the MMSE as per the relevant regulations, provided the data points for our meta‐analytic synthesis.

## Data Availability

The data supporting the findings of this study are available from the corresponding author upon reasonable request.
