# Supplementary material for: The Therapeutic Effect of Electroconvulsive Therapy on Depressive Patients of Different Ages: A Systematic Review and Meta‐Analysis
Source: Depress Anxiety. 2026 Aug 29;2026:4345722. doi: 10.1155/da/4345722 (PMC13525544; doi:10.1155/da/4345722)
Supplement: Supplementary file 1 [file DA-2026-4345722-s001.docx]

Funnel plots and Egger’s test results are as follows. These findings may suggest that the meta-analysis is unlikely to be affected by publication bias.

Figure 1 Funnel plots and Egger’s test for HAMA

Figure 2 Funnel plots and Egger’s test for the cohort studies of HAMD (13-18 and 18-60 years old)

Figure 3 Funnel plots and Egger’s test for the cohort studies of HAMD (18-60 and ＞60 years old)

Figure 4 Funnel plots and Egger’s test for the before-after-treatment studies of HAMD

Figure 5 Funnel plots and Egger’s test for the cohort studies of MADRS

Figure 6 Funnel plots and Egger’s test for the before-after-treatment studies of MADRS

Figure 7 Funnel plots and Egger’s test for MMSE

Figure 8 Funnel plots and Egger’s test for WMS-Digit span

Figure 9 Funnel plots and Egger’s test for WMS-Association

Figure 10 Funnel plots and Egger’s test for WMS-Picture

Figure 11 Funnel plots and Egger’s test for WMS-Recognition
